# Utilization of—Omic technologies in cold climate hydrocarbon bioremediation: a text-mining approach

**DOI:** 10.3389/fmicb.2023.1113102

**Published:** 2023-06-16

**Authors:** Kristopher Abdullah, Daniel Wilkins, Belinda C. Ferrari

**Affiliations:** ^1^Faculty of Science, School of Biotechnology and Biomolecular Sciences, University of New South Wales, Sydney, NSW, Australia; ^2^Environmental Stewardship Program, Australian Antarctic Division, Department of Climate Change, Energy, Environment and Water, Kingston, TAS, Australia

**Keywords:** metagenomics, proteomics, transcriptomics, text-mining, Antarctic Soils, diesel biodegradation

## Abstract

Hydrocarbon spills in cold climates are a prominent and enduring form of anthropogenic contamination. Bioremediation is one of a suite of remediation tools that has emerged as a cost-effective strategy for transforming these contaminants in soil, ideally into less harmful products. However, little is understood about the molecular mechanisms driving these complex, microbially mediated processes. The emergence of −*omic* technologies has led to a revolution within the sphere of environmental microbiology allowing for the identification and study of so called ‘unculturable’ organisms. In the last decade, −*omic* technologies have emerged as a powerful tool in filling this gap in our knowledge on the interactions between these organisms and their environment *in vivo*. Here, we utilize the text mining software Vosviewer to process meta-data and visualize key trends relating to cold climate bioremediation projects. The results of text mining of the literature revealed a shift over time from optimizing bioremediation experiments on the macro/community level to, in more recent years focusing on individual organisms of interest, interactions within the microbiome and the investigation of novel metabolic degradation pathways. This shift in research focus was made possible in large part by the rise of *omics* studies allowing research to focus not only what organisms/metabolic pathways are present but those which are functional. However, all is not harmonious, as the development of downstream analytical methods and associated processing tools have outpaced sample preparation methods, especially when dealing with the unique challenges posed when analyzing soil-based samples.

## 1. Introduction

Petroleum Hydrocarbon (HC) contamination is a common, extensive and pervasive anthropogenic contaminant in the Arctic and Antarctic (Whyte et al., 1999; Errington et al., 2018). Incidence of HC spills in these environments is correlated with increased human activities such as tourism, scientific exploration and exploitation of natural resources, often resulting from accidental spills or past mismanagement of waste (Mohn et al., 2001; Bennett et al., 2015; Camenzuli and Freidman, 2015). In Antarctica, terrestrial spills are often localized to permanent Antarctic bases and range from small localized spills at re-fueling sites through to larger spill incidents from storage tanks which can affect hundreds of square meters up to a few square kilometers of soil (Tin et al., 2009; Jesus et al., 2015). In the Arctic an estimated 42% of structures built upon permafrost are at risk due to permafrost thawing (Ramage et al., 2021), the implications of this within the context of hydrocarbon spills can be seen in events such as the 2020 collapse of a diesel storage tank due to melting permafrost, which released 21,000 tonnes of diesel into surrounding waterways including the Ambarnaya river (Glanville et al., 2020). Given the enormity of legacy and likely future spills, particularly in the Arctic with permafrost vulnerable infrastructure (Glanville et al., 2020; Ramage et al., 2021), the requirement to mitigate environmental damage through remediation and clean-up is vital.

Unlike in more temperate climates where natural attenuation of hydrocarbon spills is more rapid, terrestrial hydrocarbon spills in cold climates can persist in the environment for decades (Revill et al., 2007). While the toxicity of hydrocarbon pollutants has been shown to decline with age, ecotoxicology tests have demonstrated that contaminants from Antarctic diesel can impact the health of invertebrates even after extended aging (Brown et al., 2016, 2023). The persistence and toxicity of hydrocarbons as a pollutant in otherwise relatively remote and pristine locations makes the remediation of these contaminants a matter of import, for continued social licence to conduct scientific and other endeavors in these unique environments. The most common and effective method applied in cold regions utilizes endemic microorganisms in a process known as bioremediation (Whyte et al., 1999; Mohn et al., 2001; Josie et al., 2014; Camenzuli and Freidman, 2015; van Dorst et al., 2021). Bioremediation projects vary in sophistication and labor costs on a spectrum, from natural attenuation (effectively ‘sit back’ and monitor), to *in situ* treatment, or excavation and treatment of the soil in landfarms or engineered biopiles, in which heat, water availability, nutrient content and microbial composition can be monitored and controlled (Ruberto et al., 2003; Bento et al., 2005; Kauppi et al., 2011; Gutiérrez et al., 2020; Johnsen et al., 2021; van Dorst et al., 2021).

On site bioremediation can have lower economic and environmental costs compared to offsite disposal or treatment. This has made bioremediation an attractive option for the clean-up of hydrocarbon spills in cold climates (Ruberto et al., 2003; Martínez Álvarez et al., 2015; Errington et al., 2018). However, cold desert climates such as in the Arctic and Antarctic also pose unique challenges in the bioremediation of hydrocarbons. The extreme low temperatures and short summers reduce the volatization and bioavailability of hydrocarbons in the soil (Pawar, 2012; Gomez and Sartaj, 2013; Cipullo et al., 2019). In addition to cold temperatures, essential resources such as nitrogen, phosphorus and water are scarce, all resulting in a comparatively low microbial load and activity. Relative to temperate environments and controlled laboratory conditions, natural attenuation rates are negligible under these harsh conditions (Pawar, 2012; Gomez and Sartaj, 2013; Josie et al., 2014; Cipullo et al., 2019).

*Omic* technologies have emerged as powerful tools in untangling how native microbes respond to these unique challenges, as well as how they respond to rapid changes brought about by bioremediation processes themselves (Gupta et al., 2020; Josie et al., 2014; Ramírez-Fernández et al., 2021; Zhang et al., 2022). Antarctic soils are dominated by microbial communities, making up the majority of local genetic diversity and driving major geochemical cycles (Ruberto et al., 2009; van Dorst et al., 2021). Technologies such as 16S community profiling, metagenomics, proteomics and transcriptomics enable researchers to monitor the composition and function of microbial communities both in their natural state as well as once impacted by change (Eckford et al., 2002; Han, 2013; Magalhães et al., 2014; Simas et al., 2015). Given the objective of bioremediation projects is to reduce environmental harm, by removing contaminants like hydrocarbons (HC) from the soil without causing further damage and disruption, understanding the metabolic pathways responsible for HC degradation as well as other key nutrient cycling pathways is crucial in developing effective bioremediation strategies (Josie et al., 2014; Gupta et al., 2020; Ramírez-Fernández et al., 2021; Zhang E. et al., 2021).

## 2. Methods

The corpus used for this study was generated with Scopus search using the keywords ‘bioremediation AND (Antarctica OR ARCTIC OR COLD) AND hydrocarbons’ which yielded 104 research articles. After omitting articles related to heavy metals and other xenobiotics, 71 articles were selected. Three additional searches were conducted in which the term Antarctica was replaced with ‘genomic’, ‘proteomic’ or ‘transcriptomic’. The search period was restricted to 2002 and 2022. The articles were filtered for relevance with articles focusing on heavy metals and other xenobiotics omitted as well as review articles. In total, 117 articles were added to a Mendeley library before importing to VOSviewer version 1.6.17 (van Eck and Waltman, 2011).

## 3. Text mining

Academic papers are being produced at an unprecedented rate, with annually published research articles increasing exponentially (Fire and Guestrin, 2019). Currently, most of this information is in the form of written paragraphs otherwise known as unstructured data (Bajocco et al., 2019). Unstructured data while convenient for the reader, is often incompatible with current statistical and analytical methods, making unbiased and accurate assessment of trends within the literature difficult (Rao, 2003). The sheer volume of available information limits the capacity of human researchers to sift through the literature in a timely and accurate manner. To solve this emerging issue a variety of software capable of automatic categorization and information extraction of text data have emerged (Ganino et al., 2018; S. H. H. Shah et al., 2020). These processes commonly result in a form of text analysis known as text mining, with the text broken up or ‘tokenised’ to uncover the prevalence of key terms or phrases (Bajocco et al., 2019; Parkavi et al., 2020; Wong et al., 2021). Here, we aim to utilize text mining to uncover trends in bioremediation research, particularly related to the utilization of ‘omic’ technologies within this field.

**Figure 1:**
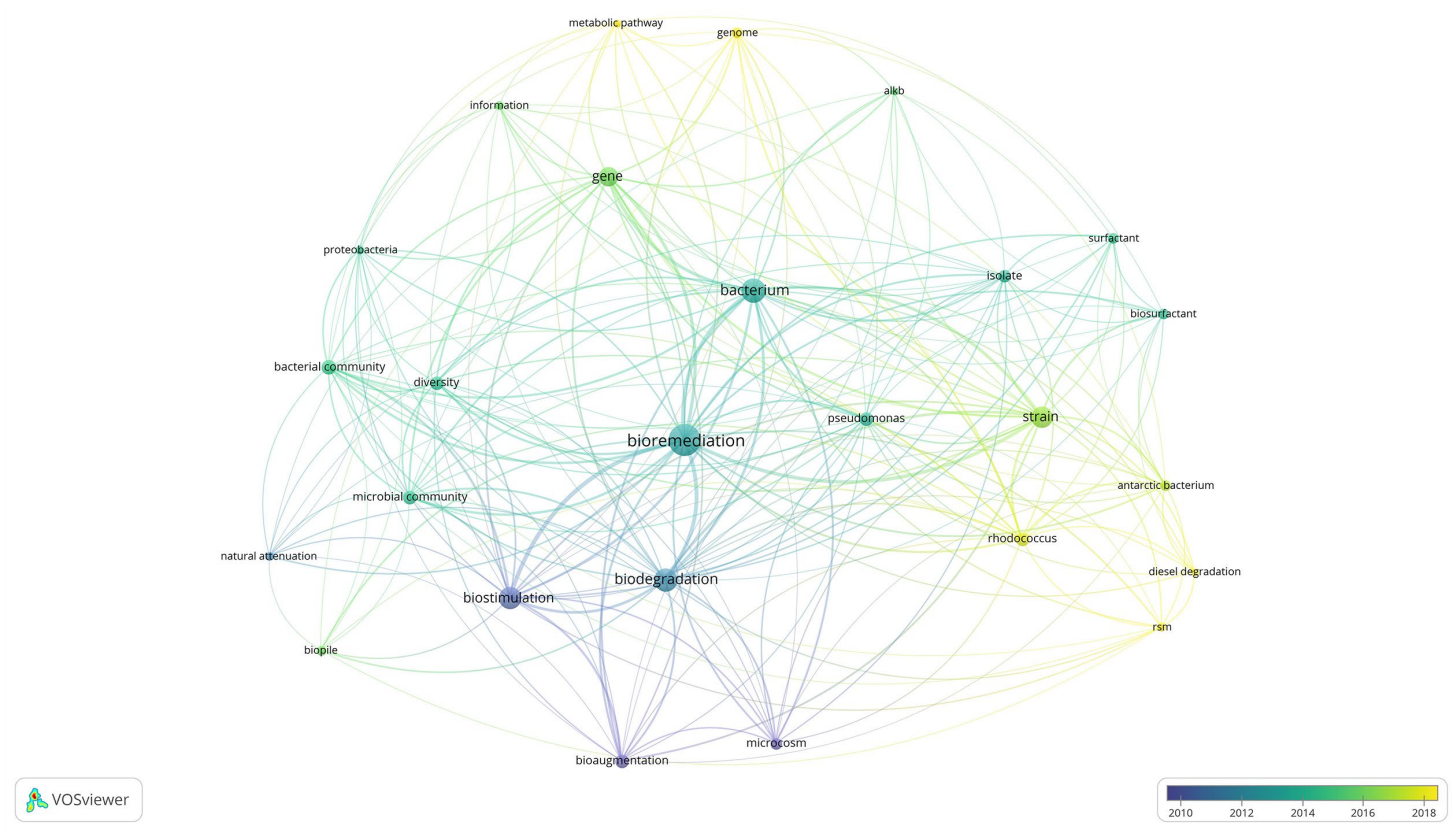
Visual network analysis of keyword frequency overlayed by average publication date.

Figure 1 displays a shift in bioremediation research from 2002 to 2022, during this time the most popular lines of inquiry shifted from a broader macroscopic level approach represented by blue and green network links to highly specific molecular scale approaches represented in yellow. Demonstrated by the shift in research focus from bioremediation and biostimulation in field and microcosm experiments, dealing with temperature, water and nutrient amendment, to sophisticated ‘omic’ studies investigating novel metabolic pathways and functional genes associated with biodegradation (Delille et al., 2004; Bento et al., 2005; Dias et al., 2015; Kai et al., 2020; Wang et al., 2021). The most prominent terms with an average publication year between 2010 and 2012 were *biostimulation* and *bioaugmentation*. In contrast, 2013–2018 focused on microbial community composition, and the function and production of metabolites of interest, such as biosurfactants. Of the commonly mentioned genera, *Rhodococcus* garnered more attention in recent years with emerging studies using metagenomics to study metabolic pathways. In Figure 1 the literature visualization tool VosViewer was utilized to provide an overview of the trends within bioremediation research over the past 20 years. There are multiple benefits to text mining approaches, the first being one of time saving time, as it provides the user with the ability to generate a visual summary of hundreds of publications instantly. The side by side comparison of decades of research may provide the stimulus for revisiting techniques that have fallen out of focus, such as bioaugmentation, in an attempt to explain the molecular mechanisms causing introduced bacteria to have limited success in field scale bioaugmentation studies (Bento et al., 2005; Stallwood et al., 2005; Ruberto et al., 2009, 2010; Kauppi et al., 2011; Watahiki et al., 2019).

## 4. Omic tools in bioremediation research

Prior to the emergence of ‘omic’ approaches the study of microbial diversity and function was limited to those organisms that were culturable (Aislabie et al., 1998; Jamal and Penninckx, 1999; Whyte et al., 1999). Consequently, a large portion of the microbial population in any given sample was ignored (Paul, 2022). Culture independent technologies such as amplicon sequencing, metagenomics, transcriptomics and proteomics have become ubiquitous in modern microbiology for their ability to produce accurate, high throughput data on microbial taxonomy, function and phylogeny (Gupta et al., 2020; Paul, 2022). The presence of ‘omic’ tools within the sphere of bioremediation in all climates is summarized in Table 1. While the bulk of the studies incorporating ‘omics’ were not focused on cold climates specifically, the summary demonstrates the potential impact of these technologies in assessing the function and composition of the local microbiome as a means of assessing soil health. Of the above mentioned ‘omic’ technologies, 16S rRNA amplicon sequencing has cemented its place as the workhorse of microbiology research (Evans et al., 2004; Païssé et al., 2010; Kauppi et al., 2011; Zhang and Lo, 2015; Koshlaf et al., 2019; van Dorst et al., 2021).

**Table 1:** Overview of utilization of omic technologies in bioremediation research.

Technology	Benefits	Disadvantages	Use in context	Reference
16S amplicon sequencing	Cheap Well established bioinformatic pipelines Can relatively quickly generate high throughput data Provides insight on microbial diversity and abundance	Does not provide insight into microbiome activity	Provides qualitative and quantitative taxonomic data. Identified the loss of diversity associated with some biostimulation protocols and HC contamination.	von Wintzingerode et al. (2002), Païssé et al. (2010), Ruberto et al. (2010), Kauppi et al. (2011), and van Dorst et al. (2020)
Metagenomics	Can be used to generate metagenome assembled genomes (MAGS) Provides insight into taxonomy and functional genes	More expensive More challenging from a bioinformatic standpoint	Construction of MAGS, access to functional genes allows for the prediction of metabolic pathways within a microbial consortium.	Khomenkov et al. (2008), Zhang et al. (2019), Dell’Anno et al. (2021), van Dorst et al. (2021), and Gao et al. (2021)
Transcriptomics	Provides information on microbiome activity Target RNA sequences can be amplified	RNA is delicate and requires precise handling Many post transcriptional changes can take place meaning transcriptomic data is not directly correlative with function/protein synthesis	Used to determined gene expression independent of population size. Used to demonstrate upregulation of genes associated with biodegradation in *Novosphinobium* while it remained in a non-growing state.	Fida et al. (2017) and Lamas et al. (2019)
Proteomics	Most accurate measure of organism function Proteins are quite stable therefore loss of data less likely in sample transport and storage	Proteins arn’t amplifiable meaning getting large enough sample volumes in sparse soil can be challenging.	Proteomic data used in the design of synthetic microbial consortiums for phenanthrene degradation. Potential negative impacts on cell wall lipoproteins and extracellular proteins caused by nutrient and artificial surfactant addition	Zhao and Poh (2008), Macchi et al. (2021), and Zhang et al. (2022)

16S amplicon sequencing involves the amplification and parallel sequencing of the highly conserved 16S small sub-unit ribosomal RNA gene to differentiate organisms based on taxonomy (von Wintzingerode et al., 2002). This technology is commonly leveraged within bioremediation studies to monitor the diversity of the microbial communities as well as the shifts in community structure that occur as bioremediation progresses (Païssé et al., 2010; Ruberto et al., 2010; Kauppi et al., 2011; van Dorst et al., 2020, 2021). The relative low costs and well established downstream bioinformatic processes associated with 16S amplicon sequencing coupled with its utility lend to its current ubiquity within environmental and medical microbiology fields (Baraniecki et al., 2002; Wong et al., 2021). This technology is limited in that it does not provide direct insight into organism function, other than what is already known about the microbes that are identified (Gupta et al., 2020).

Statistical approaches, such as linear discriminant analysis (LDA) effect size (LEfSe) can fill this gap by enabling researchers to assign specific taxa as biomarkers for desirable conditions within a bioremediation project (Xue et al., 2020; Sui et al., 2023). One potential use for this form of analysis is in testing potential nutrient amendments for desirable bacterial responses while only comparing 16S data to various experimental conditions (Xue et al., 2020; Sui et al., 2023). Crocker et al. (2019) utilized LefSe analysis of 16S data obtained from sub-Arctic soils to identify the organisms that had the greatest response to chitin supplementation within the context of degrading hexahydro-1,3,5-trinitro-1,3,5-triazine and 2,4-dinitrotoluene. The findings indicated a rapid increase in hydrocarbon degradation by bacteria and fungi in the families *Cellulomonadaceae* and *Mortierellaceae* when supplemented with biochar (Crocker et al., 2019).

Furthermore, as microbial genomes become better annotated and functional genes within taxa identified, analytical methods, such as functional annotation of prokaryotic taxa (FAPROTAX) for estimating the functional capacity of a microbiome have been developed (Sansupa et al., 2021; Hou et al., 2023). In the bioremediation space, it is common to manually track the abundance of known HC degraders in order to make an inference on whether biostimulation protocols are favorable to these organisms (Gesheva et al., 2010; Gutiérrez et al., 2020; Habib et al., 2020; S. Kumar et al., 2020; L. A. M. Ruberto et al., 2005; Tribelli et al., 2018). Tools such as FAPROTAX provide evidence for this practice by assigning functional capabilities to all taxa in the sample. For example, this technology has been applied on 16 s and functional data during electrobioremediation of oil field soils to demonstrate if the diversity and abundance of organisms harboring hydrocarbon degrading genes was greater in biochar supplemented soils (Rushimisha et al., 2023). But, limitations with the application of this analytical method stem from the FAPROTAX database being created from cultured representatives for marine and freshwater samples (Jung et al., 2021). In this case, metabolic functions attributed to the aquatic cultured bacteria used to build the FAPROTAX database may not have be represented by species of the same taxa present in a hydrocarbon contaminated soil. Additionally, the database is built entirely from cultured organisms and as a result it cannot be used to identify functional genes residing within the so called ‘microbial dark matter’ (Sansupa et al., 2021). The creating of additional databases that not only represent cultured aquatic samples but also cultured and uncultured annotated genomes of soil micro-organisms would go a long way in improving this technology (Sansupa et al., 2021; Leite et al., 2022).

Amplicon sequencing has been applied both at the field and laboratory scale in bioremediation processes, with prior taxonomic screening of a community a critical first step toward developing site-specific bioremediation strategies (Ruberto et al., 2003; van Dorst et al., 2020). It was through 16S sequencing approaches that a significant loss in microbial diversity was observed in both the initial hydrocarbon contamination stage as well as with many forms of biostimulation (Evans et al., 2004; Silva-Castro et al., 2013; van Dorst et al., 2020), with the notable exception of biostimulation strategies that rely heavily on bulking agents such as pea-straw (Josie et al., 2014; Koshlaf et al., 2019). As demonstrated by van Dorst et al. (2014), the typically taxonomically heterologous Macquarie Island soils experience a significant taxonomic and functional shift when contaminated with 1,000 mg/kg total petroleum hydrocarbons (TPH), characterized by a loss of oligotrophs and ammonium oxidizers and instead heavily favoring known HC degrading microorganisms. These results provided evidence for a predictable site-specific shift in taxonomy and function when soils experience HC toxicity that was then used for monitoring field sites during active bioremediation (van Dorst et al., 2020). Monitoring community structure has played a major role in influencing our understanding of another popular bioremediation approach called bioaugmentation, the introduction of a mixed or pure culture of known hydrocarbon degraders to a contaminated site (Gomez and Sartaj, 2013). Bioaugmentation has produced promising results in lab scale studies, slightly outperforming biostimulation alone (Bento et al., 2005; Gomez and Sartaj, 2013). However, monitoring foreign strains after introduction into a microbial community has shown that in many cases the introduced consortium often fail to persist in field-scale studies, reducing its likelihood of having a significant positive impact on hydrocarbon degradation (Bento et al., 2005; Kauppi et al., 2011; Gomez and Sartaj, 2013), with Watahiki et al. (2019) reporting significant reductions of *R. jostii* in the population 6 days post-inoculation.

Targeted next generation amplicon sequencing allows for a single gene or panel of genes to be sequenced from a sample, it is the next ‘step up’ from 16S amplicon sequencing in the context of functional analysis of soil allowing for greater specificity and accuracy. For example by targeting known hydocarbon degrading genes such as alkB in addition to other functional genes of interest (Bewicke-Copley et al., 2019; Sansupa et al., 2021). Targeted amplicon sequencing of functional genes has been used to investigate the relationship between environmental conditions and genes linked with geochemical cycling. Certain genes can act as markers for specific groups of organisms and provide information beyond their primary function, for example by monitoring the abundance of NifH genes in a hydrocarbon contaminated site researchers can determine the degree of toxicity caused by HCs as nitrifiers are known to be particularly susceptible to hydrocarbon toxicity (Pudasaini et al., 2019). While the introduction of bioavailable nitrogen to a soil system whether the source be from penguin guano or biostimulation protocols has been affiliated with an increase in de-nitrifying genes, such as NirS, NirK, and NosZ (Jung et al., 2011; Ramírez-Fernández et al., 2021; van Dorst et al., 2021). However, increased abundance of a functional gene is not always an accurate indicator of metabolites being produced by an organism, for example penguin guano impacted soils are commonly associated with high levels of NO_2_ emission (Zhu et al., 2013; Wang et al., 2019). This is despite a high abundance of NosZ genes associated with these soils - which is responsible for reducing NO_2_ to nitrogen gas (Ramírez-Fernández et al., 2021). Some possible explanations for such a discrepancy are the target gene not being expressed or uneven reaction kinetics. While limited in its capacity to predict microbiome function, targeted sequencing of functional genes within the context of bioremediation has provided a rapid and cost-effective method of monitoring abundance of key functional genes, during and after bioremediation strategies have been implemented (Mills et al., 2003; Li and Yan, 2021).

Non-sequencing-based approaches such as quantitative PCR (qPCR) have emerged as a method for quantifying genes of interest, with the benefits of reduced financial and time cost than sequencing techniques (Lasa et al., 2019; Li and Yan, 2021). The trade-off for these conveniences is less discovery power with a smaller array of genes being searched for in a single run (Lasa et al., 2019; Li and Yan, 2021). Metagenomics involves the fragmentation and sequencing the entirety of the genetic material in a sample (Albertsen et al., 2013; Meziti et al., 2021). From this pool of data, metagenome assembled genomes or MAGs can be created. The assemblage of draft genomes from metagenomic data removes the need to isolate an organism in culture before sequencing its genome. Instead, whole genomes can be assembled directly from environmental samples, this reduced reliance on culturing has saved countless hours of labor as well as generating draft genomes of organisms for which are yet to be isolated and exist as microbial dark matter (Albertsen et al., 2013; Meziti et al., 2019, 2021). The generation of MAGs from metagenomes received some early criticism due to loss of accuracy from potential contamination of reads, mis-binning and potential lack of read depth and coverage (Chen et al., 2020; Waschulin et al., 2022). However, these issues have not outweighed the primary benefit of bypassing the requirement of culturing pure isolates prior to conducting molecular studies on microorganisms, to the extent that generation of MAGs has begun to out-pace isolate derived genomes (Bowers et al., 2017).

Metagenomic techniques are gaining prominence for being the ‘omic’ one stop shop. Metagenomic data can provide information on taxonomic diversity, functional potential of the microbiome and MAGs, but importantly not information regarding gene expression (Bao et al., 2017; Zhang et al., 2019; Dell’Anno et al., 2021). Within the context of bioremediation, it is rare to find an individual organism capable of degrading a xenobiotic to its terminal point, rather it is far more likely that a composite metabolic pathway is formed between several synergistic microorganisms (Khomenkov et al., 2008; Zhang et al., 2019; Gao et al., 2021). Metagenomic screening of soils and sediments is a popular method for determining the suite of organisms and specific functional genes that contribute to a metabolic pathway (Khomenkov et al., 2008; Zhang et al., 2019; Gao et al., 2021; Baek et al., 2022; Semenova et al., 2022). Gao et al. (2021) applied this technology to the bioremediation of hydrocarbons with the comparison of the metabolic pathways - Nitrogen and Hydrocarbon within a target soil, finding glutamine and glutamate synthase to be a key enzymes in the nitrogen cycles of HC degraders. In this instance, this new information was validated in practice as ammonium was shown to be the most efficient nitrogen supplement. Metagenomic analysis of soils, *via* prediction of metabolic pathways has the potential to inform researchers and project managers on optimal nutrient amendment. Metagenomic also offers the ability to identify bottlenecks or missing elements to a HC degradation pathway and inferring tentative predictions of metabolites that could be formed during these processes (Khomenkov et al., 2008; Zhang and Lo, 2015; Zhang et al., 2019; Gao et al., 2021; Baek et al., 2022; Ibrar and Yang, 2022).

While metagenomics has grown with the promise of circumventing the bottleneck of difficult to culture organisms within microbial studies it comes with its own restrictions. These being predominantly related to limitations the bioinformatic processing of large data sets, which commonly occurs at a slower pace than the generation of sequencing datasets (Ugarte et al., 2019; Hiseni et al., 2021; Shahroodi et al., 2022). Specifically, issues relating to limited reference database coverage, lack of integration and modularity between bioinformatic pipelines and taxonomic look up functions are all current sources of bottlenecks which can reduce accuracy of results, create difficulties upgrading and maintaining pipelines or they may simply be computationally intensive (Ugarte et al., 2019; Hiseni et al., 2021; Shahroodi et al., 2022). It has also been demonstrated that MAGs at a completeness of 95% can miss up to 50% of the variable genes in a population, that is those genes that are present in greater than 10% but less than 95% of the population (Meziti et al., 2021). While likely due to incomplete or incorrect binning of contigs, the resulting lack of sensitivity to variable genes could pose a roadblock to detecting novel variants of functional genes responsible for xenobiotic degradation (Nelson et al., 2020; Meziti et al., 2021).

While metagenomics and binning approaches provide deeper and highly specific information on the taxonomy and functional capacity of individual members within a complex microbial community, is accompanied by higher comparative costs, data burden and increased complexity of downstream bioinformatic processes (Meziti et al., 2021). These limitations are consequently restricting the analysis of large metagenomics datasets to organizations with access to high performance computing clusters, yet, even with access to specialized tools, data analysis can take multiple days to a week (Tikariha and Purohit, 2019; Silva et al., 2021; Chivian et al., 2023). Nevertheless, improvements to downstream analysis pipelines and technologies are constantly occurring and with time will likely mitigate these challenges (Meziti et al., 2019, 2021; Tikariha and Purohit, 2019; Nelson et al., 2020). Bioinformatic environments such as Kbase provide powerful platforms which enable the assembly and analysis of MAGS from initial reads as well as genome analysis such as metabolic modeling and genome annotation, while these technologies are available elsewhere the shift toward centralized tools for metagenome analysis will increase accessibility, data sharing and support which are key factors in reducing limitations to this technology (Nelson et al., 2020; Chivian et al., 2023).

Transcriptomics utilizes RNA fragments that correspond to DNA from gene coding regions, such as messenger RNA, ribosomal RNA and transfer RNA through technologies such as RNA-seq, microarray and real time PCR (Lamas et al., 2019). Within the context of bioremediation, transcriptomics is used to determine the array of functional genes being expressed at a given time (Stallwood et al., 2005; Fida et al., 2017; Tribelli et al., 2018). For example, *Novosphinobium* in a bioaugmentation system was observed to enter a viable non-cultivatable state in which its population did not increase (Fida et al., 2017). However, *Novosphinobium* continued to up-regulate genes known to be associated with hydrocarbon degradation suggesting that significant shifts in microbiome function can occur during bioremediation that cannot be identified *via* taxonomic trends alone. To date, bioremediation studies leveraging transcriptomics are limited, and have focused on xenobiotics other than hydrocarbons, as it is a powerful tool in unraveling novel metabolic pathways (Das et al., 2020; Baek et al., 2022). Within the field of hydrocarbon bioremediation, transcriptomics has been of particular use in identifying genes associated with tolerance to Polycyclic Aromatic Hydrocarbons (PAHs) (Ito et al., 2022; Su et al., 2022), as well as monitoring dynamic changes in arrays of genes associated with hydrocarbon degradation (Das et al., 2020; Wang et al., 2021).

While not as abundant within bioremediation literature as metagenomics, the use of transcriptomics is growing within bioremediation studies. Transcriptomics as a method of investigating microbial response to differing environments commonly reports differentially expressed genes in the thousands to tens of thousands (Fida et al., 2017; Tribelli et al., 2018; Das et al., 2020), which is significantly more data than other technologies such as meta-proteomics used for this purpose (Zhao and Poh, 2008; Zhang et al., 2022). Transcriptomics has been used to uncover metabolic pathways and novel gene expression shifts in an environment independent of taxonomic shifts. For example, such as during exposure of *Pseudomonas aeruginosa* to crude oil, where interestingly some HC degrading genes were shown to be downregulated like xylL, xylX, and antA in the presence of crude oil (Das et al., 2020). When considering the sheer diversity of potential and known HC degrading genes that were differentially expressed it suggests *P. aeruginosa* is capable of several pathways of HC degradation and is capable of tailoring gene expression to the form of HC in its environment (Das et al., 2020). This discovery has implications for wider studies focused on bioremediation, for example transcriptomic analysis of potential HC degraders could be useful in organism selection regarding bioaugmentation studies.

Transcriptomics also finds practical use in uncovering natural and artificial factors they may lead to suppression of key HC degrading genes, for example early findings in marine samples show greater upregulation of the alkB gene in hydrocarbon degrading bacteria supplemented with biosurfactants than with those supplemented with Ultrasperse 2 (De Couto et al., 2016). Further investigation into this area could be beneficial as utilization of chemical surfactants has not been sufficiently explored in relation to their potential toxicity and how that may affect expression of hydrocarbon degrading genes (De Couto et al., 2016).

RNA is characterized by low stability in *in-vitro* studies, the lability of RNA is increased in the context of cold environments as many psychrophilic RNAase’s can remain active at low temperatures (Cristescu, 2019; Gunjal Aparna et al., 2021; Hualpa-Cutipa et al., 2022). The other issue facing RNA analysis is the co-extraction of contaminants that can interfere with downstream enzyme applications (Tveit et al., 2014). Common solutions to these complications are centered around amplification through PCR (Rio, 2014; Tveit et al., 2014; Cristescu, 2019). It has been demonstrated that contaminants can be essentially eliminated through dilution followed by linear amplification of RNA template (Tveit et al., 2014). In contexts where the lability of RNA is a concern, such as when transporting samples back from remote locations, reverse transcription of RNA sequences to DNA can enhance the stability of samples without losing read depth (Rio, 2014).

Meta-proteomics involves the quantification of all intra and extra-cellular proteins in an environmental sample most commonly *via* high performance liquid chromatography combined with mass spectrometry (Wilmes and Bond, 2006; Manuel, 2022). In addition to quantifying structural and intra-cellular proteins, proteomics provides a more accurate view of community function than transcriptomics due to post transcriptional changes that can occur before protein synthesis (Schenk et al., 2019). Meta-proteomic analysis can benefit current understanding of bioremediation projects primarily in three ways; elucidating the effect specific biostimulants might have on enzyme activity, screening for the presence of known hydrocarbon degrading enzymes in soil and uncovering the protein expression of a known isolate (Macchi et al., 2021; Méndez García and García de Llasera, 2021; Baek et al., 2022; Zhang et al., 2022).

Certain bioremediation treatments such as the addition of chemical surfactants or biosurfactants can affect protein function independently of gene expression. For example, full proteomic analysis of *B. subtilis* revealed a negative correlation between artificial surfactants and function of proteins associated with alkane degradation, whereas bio-surfactants did not negatively impact protein function (Zhang et al., 2022). Proteomic analysis has been the predominant technology used for identifying enzymes involved in PAH degradation (Méndez García and García de Llasera, 2021). PAHs can be more persistent in the environment and often require a more complex metabolic pathway to reach terminal oxidation (Gomez and Sartaj, 2013; Cipullo et al., 2019). In a study investigating the biodegradation of toluene in a bioelectric well, meta-proteomic analysis was used to investigate protein expression in the bulk of the reactor in addition to the anode. The study revealed the abundance of proteins related to hydrocarbon degradation were skewed toward the bulk phase of the reactor, with proteins involved with the tricarboxylic acid cycle featuring prominently on the biofilm formed on the anode surface (Tucci et al., 2022). It is worth noting that the proteins identified from the anode were low, with 46 and 67 proteins identified from each trial, this is a common shortcoming of metaproteomic analysis with no means of protein amplification low protein yield from difficult samples, can significantly impact the results (Tribelli et al., 2018; Tucci et al., 2022). Nonetheless, when combined with other omic technologies such as shotgun sequencing, it is likely that novel metabolic pathways can be predicted. Recently, Tucci et al. (2022) proposed a three step electrogenic degradation pathway that involves initial digestion of toluene by anaerobic hydrocarbon degraders, the resulting unknown intermediaries are then fermented before terminal oxidation by *Geobacter* sp. residing on the anode.

One pathway to improving the degradation of PAHs is by identifying hydrocarbon degraders *via in silico* analysis which can then be confirmed *via* shotgun proteomics (Macchi et al., 2021). Furthermore, a synthetic microbial consortium designed in this way was shown to be effective at degrading phenanthrene in liquid culture, but it is unclear if the same results could be achieved in contaminated soils as past bioaugmentation studies have observed a lack of persistence of inoculate *in situ* (Bento et al., 2005; Gutiérrez et al., 2020; Macchi et al., 2021). Currently these studies select consortiums based on individual members ability to degrade a particular hydrocarbon (Baek et al., 2022). However, as understanding of microbial interactions increases, it is likely that some microbes will be added to these consortiums with the desired effect of supporting overall consortium health thereby indirectly increasing the efficiency of hydrocarbon degraders.

The bottleneck for *‘omic’* studies is often the preparation of complex environmental samples for downstream analysis. Soil DNA extraction followed by 16S amplicon sequencing or metagenomic analysis has been widely utilized, with proven sample preparation methods available that have been employed in bioremediation projects (Ferrari et al., 2016; Koshlaf et al., 2019; Gupta et al., 2020; Gao et al., 2021; van Dorst et al., 2021). However, when it comes to the study of both tRNA and proteins, many of the established methodologies have focused on analyzing concentrated cell samples comprised of a cultivated isolate only, often making them less suitable for direct application to environmental samples (Tribelli et al., 2018; Mukherjee et al., 2022). The afore mentioned trend is also present in meta-proteomic studies, with *in-vitro* meta-proteomic samples, originating from a pure culture being more widespread than samples of *ex-vitro* origins (Zhao and Poh, 2008; Schenk et al., 2019; Kumar et al., 2020; Macchi et al., 2021; Zhang E. et al., 2021). This imbalance has resulted in current methods of analyzing protein samples outstripping sample preparation methods (Zhao and Poh, 2008; Chourey et al., 2010; Schenk et al., 2019; Das et al., 2020; Macchi et al., 2021; Zhang et al., 2022).

The application of meta-proteomics to bioremediation within cold climates faces unique challenges. Low biological yields necessitate the concentration of samples, while substances, known to interfere with Gas Chromatography/Mass Spectroscopy, such as salts, DNA and humic acids are co-extracted along with proteins (Chourey et al., 2010; Tschitschko et al., 2016; Abiraami et al., 2020). These factors in combination can limit discovery power in soil metaproteomic studies (Tucci et al., 2022). It should be noted that this limitation is less prominent in aquatic environmental samples as well as *in vitro* studies (Tschitschko et al., 2016; Macchi et al., 2021), likely due to easier access to greater sample concentrations and reductions in detritus prior to any sample processing being applied (Chourey et al., 2010; Abiraami et al., 2020). The development of multiplexing protocols such as labeling peptides with isobaric tags has emerged as a potential solution to the issue of protein identification in complex samples and offers greater quantitative accuracy and discoverability (Creskey et al., 2022). The primary limitation to this technique is the associated high costs and use of volatile compounds such as acetonitrile, which need to be handled with care as degradation or inaccurate transfer can lead to inaccurate sample quantitation (Creskey et al., 2022). Currently, Tandem Mass Tag (TMT) labeling has been successfully utilized to provide a detailed insights into the molecular mechanisms involved in PAH degradation in both bacteria and yeasts (Li et al., 2021; Zhang L. et al., 2021), although it should be noted that these studies analyzed samples grown in culture and the potential for multi-plexed labeled techniques to solve the challenges posed to soil-based samples is yet to be investigated.

The optimization of sample preparation techniques for soil are required, specifically when dealing molecules such as proteins, current sample cleaning methods result in protein loss and are thus antagonistic toward the need to extract more protein, whereas by extracting more protein a greater amount of contaminants are co-extracted requiring more sample cleaning.

## 5. Insights from omic technologies in the polar environment, molecular mechanisms driving hydrocarbon biodegradation

Table 2 summarizes some of the key taxa and their HC degrading potential that *‘Omic’* research has unveiled. When applied within the context of psychrophillic, psychrotrophic and industrially exploitable organisms in cold climate, hydrocarbon contaminated soils, and their products (Ruberto et al., 2005; Parrilli et al., 2010; Kumar et al., 2020).

**Table 2:** Summary of key hydrocarbon degrading bacteria.

Taxa	HC degrading capabilities	Reference
*Pseudomonas*	Production of biosurfactants xylL and XylX mediated PAH degradation terminal alkane oxidation via AlkB initiated pathway Crude oil degradation	Das et al. (2020), Li et al. (2020), Semenova et al. (2022), and Hou et al. (2023)
*Rhodococcus*	Production of biosurfactants Terminal alkane oxidation oxidation via alkB initiated pathway	Li et al. (2020) and Semenova et al. (2022)
*Lysinibacillus*	Terminal oxidation of alkane via alkB mediated pathway	Li et al. (2020)
*Cellumonadacea*	Potential hexahydro-1,3,5-trinitro-1,3,5-triazine degrader	Crocker et al. (2019)
*Methylosinus*	Crude oil degradation	Hou et al. (2023)
*Marinobacter*		
*Rhodocyclaceae*	Anaerobic fermentation of toluene	Tucci et al. (2022)

The utilization of ‘omic’ technologies, especially regarding upstream sample preparation, is tailored toward ecological or industry focused studies. Their popularity can be accredited to their capacity to unravel the molecular mechanisms underpinning a process of interest (Jung et al., 2011; Rio, 2014; Jurelevicius et al., 2021; Méndez García and García de Llasera, 2021; Ramírez-Fernández et al., 2021; van Dorst et al., 2021). In the context of hydrocarbon bioremediation, the primary focus in the use of these technologies has been investigating the mechanisms driving alkane and PAH degradation (Jurelevicius et al., 2012; Tribelli et al., 2018; Kuc et al., 2019). When assessing the capacity of a microbiome to degrade diesel the relative abundance of a gene called alkB which encodes the alkane monooxygenase enzyme is used (Yergeau et al., 2012; Crane et al., 2018; Li et al., 2020; Ling et al., 2023). Alkane monooxygenase is the first enzyme in the pathway responsible for the terminal oxidation of alkanes and has been commonly found in hydrocarbon contaminated soil in both cold and temperate climates (Yergeau et al., 2012; Crane et al., 2018; Li et al., 2020; Ling et al., 2023). Within the context of bioremediation research, an increase in alkB is thought to be associated with the degradation of short chain alkanes, which form a large proportion by mass of Antarctic blend diesels (Yergeau et al., 2012; Kuc et al., 2019; Semenova et al., 2022). Within hydrocarbon contaminated soils it is not uncommon to find an alkB relative abundance of greater than 100 gene copies detected per 100 organisms, likely due to many organisms having multiple copies of this gene (Yergeau et al., 2012). Due to this confounding variable, it may not be suitable to use alkB abundance in a population as the sole indicator for alkane degradation potential in aerobic soils. Instead a more reliable array of molecular indicators can be assembled from the identification of a significant population of known hydrocarbon degraders. For example targeting *Rhodococcus* and *Pseudomonas via* amplicon sequencing (Gutiérrez et al., 2020; Kai et al., 2020), who harbor and abundance of alk and acetyl-CoA synthase genes which are indicators of the alkane oxidation pathway reaching its terminus (Li et al., 2020; Semenova et al., 2022).

The above mentioned functional genes and enzymes have been frequently demonstrated to correlate with hydrocarbon degradation rate and ‘completeness’, therefore it may be viable to screen for their abundance as an indicator of microbiome ‘fitness’ within the context of degrading hydrocarbons (Yergeau et al., 2012; Crane et al., 2018; Li et al., 2020; Ling et al., 2023). Understanding the degradation pathways of PAHs is of particular importance, analysis of functional genes in both cold and temperate soils report co-metabolism of PAHs by numerous members of the microbiome more commonly than terminal catabolism by a single organism (Muangchinda et al., 2015; Li et al., 2020; Ali et al., 2023; Sui et al., 2023). Greater diversity among the structure and potential toxicity of PAHs leads to greater complexity among the molecular mechanisms underpinning their degradation (Muangchinda et al., 2015; Li et al., 2020; Ali et al., 2023; Sui et al., 2023). In a study investigating the impact of contamination of native soil from a temperate climate with two PAHs, benzene and benzo[a]pyrene (BaP) demonstrated the increased biodegradation of BaP while co-contaminated with benzene compared to when contaminated with BaP alone, inversely the degradation of benzene occurred faster when it was the sole contaminant (Ali et al., 2023). This result is likely due to the composite nature of metabolic pathways for PAH degradation. This phenomenon has also been observed at the level of an individual organisms, such as *L. fusiformis* when cultured in isolation in petroleum contaminated soil. Here, the upregulation of alkB and acetyl-coA synthase was demonstrated suggesting the capacity for terminal oxidation of alkanes (Li et al., 2020). At the same time the upregulation of enzymes that catalyze the oxidation of aromatic compounds such as cyclohexanone monooxygenase was observed (Li et al., 2020). Interestingly, *L. fusiformis* did not appear to express cytochrome P450 alkane hydroxylase which is associated with the oxidation of medium chain alkanes, this finding was further evidenced by reduced growth rates when diesel consisting of medium chain alkanes was used as a carbon source (Li et al., 2020).

As biostimulation protocols can be associated with a loss of community diversity, there is a case to be made that with lower diversity some steps in the co-metabolic pathway of PAH degradation may also be lost (Evans et al., 2004; Cury et al., 2015; Van Goethem et al., 2020). However, metagenomic analysis of soil communities in both temperate and cold hydrocarbon contaminated soils have reported a high degree of redundancy among PAH degrading genes (Jurelevicius et al., 2012; Muangchinda et al., 2015; Ali et al., 2023; Lv et al., 2023). In cold climates, little is known about the degradation pathways used by anaerobic hydrocarbon degraders despite evidence of their being present in contaminated soils with high organic carbon on King George Island (Sampaio et al., 2017). However, anaerobic degradation of the HCs toulene and hexadecane, as mediated by benzoyl-CoA reducatase was shown to be more effective than aerobic processes in a biostimulated microcosm study using spiked soils from Casey station, Antarctica (Powell et al., 2006). Despite these promising findings, anaerobic HC degradation mechanisms in cold climates is understudied, this gap in the literature is likely due to the utility of biopiles for the degradation of hydrocarbons in these climates; slower contaminant oxidation rates and higher O_2_ saturation in many cold climate soils are all factors which highly favor aerobic processes (Delille and Coulon, 2008; Whelan et al., 2015; Martínez Álvarez et al., 2017; van Dorst et al., 2021). In contrast, anaerobic processes are better studied in warmer climates where methanogens have been observed degrading HCs through fermentative processes (Liu et al., 2019; Madison et al., 2023). Enzymes such as naphthyl-2-methyl-succinate synthase, naphthalene carboxylase, alkyl succinate synthase, and benzoyl coenzyme A have been shown to be significantly upregulated in anaerobic HC contaminated soils (Liu et al., 2019; Madison et al., 2023). Many of these enzymes are associated with PAH degradation, offering a potential explanation for observations that these organisms outperform aerobic bacteria in degrading larger alkanes and PAHs (Cason et al., 2019; Liu et al., 2019; Madison et al., 2023).

## 6. Conclusion

Text mining is an effective method for generating a visual overview of the current literature but selection, as well as extrapolation of information from keywords requires careful consideration. The emergence of *omic* technologies have revolutionized microbiology and as they become more widely available, multi-omic studies will enable a more complete picture of the molecular landscape. Significant progress has been made in mapping out co-metabolic pathways associated with the aerobic degradation of hydrocarbons in cold climates and biomarkers such as AlkB and acetyl-CoA have been shown to correlate with terminal oxidation of alkanes. However, anerobic processes in cold climates are still not well understood, although there is evidence that they could be utilized for the preferential degradation of PAHs. Although great strides have been made, several barriers remain before these technologies can be truly effective as a monitoring tool within the context of bioremediation. Three key impediments are the high costs of the more sophisticated metagenomics and transcriptomic approaches, the specialized facilities required for down-stream analysis, and difficulty with upstream processing of soil samples. Moving forward, improvements in sample preparation that address the issues of rapid sample degradation, potentially in the form of more cost effective DNAase, RNAase and proteinase inhibitors, and co-extraction of potential contaminating molecules while reducing sample loss will greatly benefit the utilization of meta-proteomics and meta-transcriptomics within the context of environmental samples. In the future decreasing costs of multi-omic studies will enable a deeper understanding on how microorganisms interact with hydrocarbons, other xenobiotics and their environment.

## Author contributions

BF was determined the theme and direction of this article with input from KA. KA generated the figures and tables. BF, DW, and KA wrote the manuscript. All Authors contributed to the article and approved the submitted version.

## Funding

This work was supported by the Australian Government research training program (RTP) scholarship awarded to KA and an Australian Research Council Future Fellowship (FT170100341) grant awarded to BF.

## Conflict of interest

The authors declare that the research was conducted in the absence of any commercial or financial relationships that could be construed as a potential conflict of interest.

## Publisher’s note

All claims expressed in this article are solely those of the authors and do not necessarily represent those of their affiliated organizations, or those of the publisher, the editors and the reviewers. Any product that may be evaluated in this article, or claim that may be made by its manufacturer, is not guaranteed or endorsed by the publisher.

## References

[ref1] Abiraami T. V. Singh S. Nain L. (2020). Soil metaproteomics as a tool for monitoring functional microbial communities: promises and challenges. Rev. Environ. Sci. Biotechnol. 19, 73–102. doi: 10.1007/s11157-019-09519-8

[ref2] Aislabie J. McLeod M. Fraser R. (1998). Potential for biodegradation of hydrocarbons in soil from the Ross Dependency, Antarctica. Appl. Microbiol. Biotechnol. 49, 210–214. doi: 10.1007/s002530051160

[ref3] Albertsen M. Hugenholtz P. Skarshewski A. Nielsen K. L. Tyson G. W. Nielsen P. H. (2013). Genome sequences of rare, uncultured bacteria obtained by differential coverage binning of multiple metagenomes. Nat. Biotechnol. 31, 533–538. doi: 10.1038/nbt.2579, PMID: 23707974

[ref4] Ali M. Song X. Wang Q. Zhang Z. Che J. Chen X.. (2023). Mechanisms of biostimulant-enhanced biodegradation of PAHs and BTEX mixed contaminants in soil by native microbial consortium. Environ. Pollut. 318:120831. doi: 10.1016/j.envpol.2022.120831, PMID: 36509345

[ref5] Baek J. H. Kim K. H. Lee Y. Jeong S. E. Jin H. M. Jia B.. (2022). Elucidating the biodegradation pathway and catabolic genes of benzophenone-3 in Rhodococcus sp. S2-17. Environ. Pollut. 299:118890. doi: 10.1016/j.envpol.2022.118890, PMID: 35085657

[ref6] Bajocco S. Raparelli E. Teofili T. Bascietto M. Ricotta C. (2019). Text mining in remotely sensed phenology studies: a review on research development, main topics, and emerging issues. Remote Sens. 11:2751. doi: 10.3390/rs11232751

[ref7] Bao Y.-J. Xu Z. Li Y. Yao Z. Sun J. Song H. (2017). High-throughput metagenomic analysis of petroleum-contaminated soil microbiome reveals the versatility in xenobiotic aromatics metabolism. J. Environ. Sci. 56, 25–35. doi: 10.1016/j.jes.2016.08.022, PMID: 28571861

[ref8] Baraniecki C. A. Aislabie J. Foght J. M. (2002). Characterization of Sphingomonas sp. Ant 17, an aromatic hydrocarbon-degrading bacterium isolated from Antarctic soil. Microb. Ecol. 43, 44–54. doi: 10.1007/s00248-001-1019-3, PMID: 11984628

[ref9] Bennett J. R. Shaw J. D. Terauds A. Smol J. P. Aerts R. Bergstrom D. M.. (2015). Polar lessons learned: long-term management based on shared threats in Arctic and Antarctic environments. Front. Ecol. Environ. 13, 316–324. doi: 10.1890/140315

[ref10] Bento F. M. Camargo F. A. O. Okeke B. C. Frankenberger W. T. (2005). Comparative bioremediation of soils contaminated with diesel oil by natural attenuation, biostimulation and bioaugmentation. Bioresour. Technol. 96, 1049–1055. doi: 10.1016/j.biortech.2004.09.008, PMID: 15668201

[ref11] Bewicke-Copley F. Arjun Kumar E. Palladino G. Korfi K. Wang J. (2019). Applications and analysis of targeted genomic sequencing in cancer studies. Comput. Struct. Biotechnol. J. 17, 1348–1359. doi: 10.1016/j.csbj.2019.10.004, PMID: 31762958 PMC6861594

[ref12] Bowers R. M. Kyrpides N. C. Stepanauskas R. Harmon-Smith M. Doud D. Reddy T. B. K.. (2017). Minimum information about a single amplified genome (MISAG) and a metagenome-assembled genome (MIMAG) of bacteria and archaea. Nat. Biotechnol. 35, 725–731. doi: 10.1038/nbt.3893, PMID: 28787424 PMC6436528

[ref13] Brown K. E. King C. K. Kotzakoulakis K. George S. C. Harrison P. L. (2016). Assessing fuel spill risks in polar waters: Temporal dynamics and behaviour of hydrocarbons from Antarctic diesel, marine gas oil and residual fuel oil. Mar. Pollut. Bull. 110, 343–353. doi: 10.1016/j.marpolbul.2016.06.042, PMID: 27389459

[ref14] Brown K. E. Wasley J. King C. K. (2023). Assessing risks from fuel contamination in Antarctica: Dynamics of diesel ageing in soil and toxicity to an endemic nematode. Ecotoxicol. Environ. Saf. 249:114345. doi: 10.1016/j.ecoenv.2022.114345, PMID: 36508834

[ref15] Camenzuli D. Freidman B. L. (2015). On-site and in situ remediation technologies applicable to petroleum hydrocarbon contaminated sites in the antarctic and arctic. Polar Res. 34, 69–77. doi: 10.3402/polar.v34.24492

[ref16] Cason E. D. Vermeulen J.-G. Müller W. J. van Heerden E. Valverde A. (2019). Aerobic and anaerobic enrichment cultures highlight the pivotal role of facultative anaerobes in soil hydrocarbon degradation. J. Environ. Sci. Health A 54, 408–415. doi: 10.1080/10934529.2018.1558902, PMID: 30676291

[ref17] Chen L.-X. Anantharaman K. Shaiber A. Eren A. M. Banfield J. F. (2020). Accurate and complete genomes from metagenomes. Genome Res. 30, 315–333. doi: 10.1101/gr.258640.119, PMID: 32188701 PMC7111523

[ref18] Chivian D. Jungbluth S. P. Dehal P. S. Wood-Charlson E. M. Canon R. S. Allen B. H.. (2023). Metagenome-assembled genome extraction and analysis from microbiomes using KBase. Nat. Protoc. 18, 208–238. doi: 10.1038/s41596-022-00747-x, PMID: 36376589

[ref19] Chourey K. Jansson J. VerBerkmoes N. Shah M. Chavarria K. L. Tom L. M.. (2010). Direct cellular Lysis/Protein extraction protocol for soil metaproteomics. J. Proteome Res. 9, 6615–6622. doi: 10.1021/pr100787q, PMID: 20954746

[ref20] Cipullo S. Nawar S. Mouazen A. M. Campo-Moreno P. Coulon F. (2019). Predicting bioavailability change of complex chemical mixtures in contaminated soils using visible and near-infrared spectroscopy and random forest regression. Sci. Rep. 9:4492. doi: 10.1038/s41598-019-41161-w 30872800 PMC6418180

[ref21] Crane S. L. van Dorst J. Hose G. C. King C. K. Ferrari B. C. (2018). Microfluidic qPCR enables high throughput quantification of microbial functional genes but requires strict curation of primers. Front. Environ. Sci. 6:145. doi: 10.3389/fenvs.2018.00145

[ref22] Creskey M. Li L. Ning Z. Fekete E. E. F. Mayne J. Walker K.. (2022). An economic and robust TMT labeling approach for high throughput proteomic and metaproteomic analysis. Proteomics:2200116. doi: 10.1002/pmic.202200116 36528842

[ref23] Cristescu M. E. (2019). Can environmental RNA revolutionize biodiversity science? Trends Ecol. Evol. 34, 694–697. doi: 10.1016/j.tree.2019.05.003, PMID: 31160082

[ref24] Crocker F. H. Jung C. M. Indest K. J. Everman S. J. Carr M. R. (2019). Effects of chitin and temperature on sub-Arctic soil microbial and fungal communities and biodegradation of hexahydro-1,3,5-trinitro-1,3,5-triazine (RDX) and 2,4-dinitrotoluene (DNT). Biodegradation 30, 415–431. doi: 10.1007/s10532-019-09884-9 31250271

[ref25] Cury J. C. Jurelevicius D. A. Villela H. D. M. Jesus H. E. Peixoto R. S. Schaefer C. E. G. R.. (2015). Microbial diversity and hydrocarbon depletion in low and high diesel-polluted soil samples from Keller Peninsula, South Shetland Islands, Antarctic. Science 27, 263–273. doi: 10.1017/S0954102014000728

[ref26] Das D. Mawlong G. T. Sarki Y. N. Singh A. K. Chikkaputtaiah C. Boruah H. P. D. (2020). Transcriptome analysis of crude oil degrading *Pseudomonas aeruginosa* strains for identification of potential genes involved in crude oil degradation. Gene 755:144909. doi: 10.1016/j.gene.2020.144909, PMID: 32569720

[ref27] De Couto C. R. A. Jurelevicius D. A. Alvarez V. M. van Elsas J. D. Seldin L. (2016). Response of the bacterial community in oil-contaminated marine water to the addition of chemical and biological dispersants. J. Environ. Manag. 184, 473–479. doi: 10.1016/j.jenvman.2016.10.039, PMID: 28314395

[ref28] Delille D. Coulon F. (2008). Comparative mesocosm study of biostimulation efficiency in two different oil-amended sub-antarctic soils. Microb. Ecol. 56, 243–252. doi: 10.1007/s00248-007-9341-z, PMID: 18074169

[ref29] Delille D. Coulon F. Pelletier E. (2004). Biostimulation of natural microbial assemblages in oil-amended vegetated and desert sub-antarctic soils. Microb. Ecol. 47, 407–415. doi: 10.1007/s00248-003-2024-5, PMID: 14681739

[ref30] Dell’Anno F. van Zyl L. J. Trindade M. Brunet C. Dell’Anno A. Ianora A.. (2021). Metagenome-assembled genome (MAG) of Oceancaulis alexandrii NP7 isolated from Mediterranean Sea polluted marine sediments and its bioremediation potential. G3 Genes|Genomes|Genetics 11:jkab210. doi: 10.1093/g3journal/jkab210 34544124 PMC8496225

[ref31] Dias R. L. Ruberto L. Calabró A. Balbo A. L. Del Panno M. T. Mac Cormack W. P. (2015). Hydrocarbon removal and bacterial community structure in on-site biostimulated biopile systems designed for bioremediation of diesel-contaminated Antarctic soil. Polar Biol. 38, 677–687. doi: 10.1007/s00300-014-1630-7

[ref32] Eckford R. Cook F. D. Saul D. Aislabie J. Foght J. (2002). Free-Living heterotrophic nitrogen-fixing bacteria isolated from fuel-contaminated Antarctic Soils. Appl. Environ. Microbiol. 68, 5181–5185. doi: 10.1128/AEM.68.10.5181-5185.2002, PMID: 12324373 PMC126386

[ref33] Errington I. King C. K. Wilkins D. Spedding T. Hose G. C. (2018). Ecosystem effects and the management of petroleum-contaminated soils on subantarctic islands. Chemosphere 194, 200–210. doi: 10.1016/j.chemosphere.2017.11.157, PMID: 29207352

[ref34] Evans F. F. Rosado A. S. Sebastián G. V. Casella R. Machado P. L. O. A. Holmström C.. (2004). Impact of oil contamination and biostimulation on the diversity of indigenous bacterial communities in soil microcosms. FEMS Microbiol. Ecol. 49, 295–305. doi: 10.1016/j.femsec.2004.04.007, PMID: 19712422

[ref35] Ferrari B. C. Bissett A. Snape I. van Dorst J. Palmer A. S. Ji M.. (2016). Geological connectivity drives microbial community structure and connectivity in polar, terrestrial ecosystems. Environ. Microbiol. 18, 1834–1849. doi: 10.1111/1462-2920.13034, PMID: 26310523

[ref36] Fida T. T. Moreno-Forero S. K. Breugelmans P. Heipieper H. J. Röling W. F. M. Springael D. (2017). Physiological and Transcriptome Response of the Polycyclic Aromatic Hydrocarbon Degrading Novosphingobium sp. LH128 after Inoculation in Soil. Environ. Sci. Technol. 51, 1570–1579. doi: 10.1021/acs.est.6b03822, PMID: 28040887

[ref37] Fire M. Guestrin C. (2019). Over-optimization of academic publishing metrics: Observing Goodhart’s Law in action. GigaScience 8:giz053. doi: 10.1093/gigascience/giz053, PMID: 31144712 PMC6541803

[ref38] Ganino G. Lembo D. Mecella M. Scafoglieri F. (2018). Ontology population for open-source intelligence: A GATE-based solution. Software Prac. Exp. 48, 2302–2330. doi: 10.1002/spe.2640

[ref39] Gao Y. Du J. Bahar M. M. Wang H. Subashchandrabose S. Duan L.. (2021). Metagenomics analysis identifies nitrogen metabolic pathway in bioremediation of diesel contaminated soil. Chemosphere 271:129566. doi: 10.1016/j.chemosphere.2021.129566, PMID: 33460896

[ref40] Gesheva V. Stackebrandt E. Vasileva-Tonkova E. (2010). Biosurfactant production by halotolerant *rhodococcus fascians* from Casey Station, Wilkes Land, antarctica. Curr. Microbiol. 61, 112–117. doi: 10.1007/s00284-010-9584-7, PMID: 20135319

[ref41] Glanville H. Cage A. Law A. (2020). A 20,000-tonne oil spill is contaminating the Arctic – it could take decades to clean up. The Conversation. Available at: https://theconversation.com/a-20-000-tonne-oil-spill-is-contaminating-the-arctic-it-could-take-decades-to-clean-up-141264 (Accessed October 28, 2022).

[ref42] Gomez F. Sartaj M. (2013). Field scale ex-situ bioremediation of petroleum contaminated soil under cold climate conditions. Int. Biodeterior. Biodegradation 85, 375–382. doi: 10.1016/j.ibiod.2013.08.003

[ref43] Gunjal Aparna B. WaghmodeMeghmala S. Patil Neha N. (2021). Role of extremozymes in bioremediation. Res. J. Biotechnol. 16:3. Available at: https://www.researchgate.net/publication/342821710_Role_of_Extremozymes_in_Bioremediation_A_Review

[ref44] Gupta K. Biswas R. Sarkar A. (2020). “Advancement of Omics: Prospects for Bioremediation of Contaminated Soils” in Microbial Bioremediation & Biodegradation. ed. Shah M. P. (Singapore: Springer Singapore), 113–142.

[ref45] Gutiérrez E. J. Abraham M. D. R. Baltazar J. C. Vázquez G. Delgadillo E. Tirado D. (2020). *Pseudomonas fluorescens*: A bioaugmentation strategy for oil-contaminated and nutrient-poor soil. Int. J. Environ. Res. Public Health 17:6959. doi: 10.3390/ijerph17196959, PMID: 32977570 PMC7579645

[ref46] Habib S. Ahmad S. A. Johari W. L. W. Shukor M. Y. A. Alias S. A. Smykla J.. (2020). Production of lipopeptide biosurfactant by a hydrocarbon-degrading antarctic rhodococcus. Int. J. Mol. Sci. 21, 1–21. doi: 10.3390/ijms21176138 PMC7504157 32858859

[ref47] Han J. (2013). Short-Term Effect of Elevated Temperature on the Abundance and Diversity of Bacterial and Archaeal amoA Genes in Antarctic Soils. J. Microbiol. Biotechnol. 23, 1187–1196. doi: 10.4014/jmb.1305.05017, PMID: 23751559

[ref48] Hiseni P. Rudi K. Wilson R. C. Hegge F. T. Snipen L. (2021). HumGut: a comprehensive human gut prokaryotic genomes collection filtered by metagenome data. Microbiome 9:165. doi: 10.1186/s40168-021-01114-w 34330336 PMC8325300

[ref49] Hou Z. Zhou Q. Mo F. Kang W. Ouyang S. (2023). Enhanced carbon emission driven by the interaction between functional microbial community and hydrocarbons: an enlightenment for carbon cycle. Sci. Total Environ. 867:161402. doi: 10.1016/j.scitotenv.2023.161402, PMID: 36638996

[ref50] Hualpa-Cutipa E. Acosta R. A. S. Cariga O. J. M. Espinoza-Medina M. A. Hansen-Reyes M. Medina-Cerna D.. (2022). “Omics Insights into Cold Environments: Cold-Tolerant Microorganisms and their Potential Use in Bioremediation” in Omics Insights in Environmental Bioremediation. eds. Kumar V. Thakur I. S. (Singapore: Springer Nature Singapore), 437–453.

[ref51] Ibrar M. Yang X. (2022). Reconstructing polyaromatic hydrocarbons degrading pathways in the enriched bacterial consortium and their biosurfactants characterization. Journal of Environmental. Chem. Eng. 10:7219. doi: 10.1016/j.jece.2022.107219

[ref52] Ito K. Hano T. Ito M. Onduka T. Ohkubo N. Mochida K. (2022). Integrated transcriptomic and metabolomic analyses reveal mechanism underlying higher resistance of the marine oligochaete Thalassodrilides cf. briani (Clitellata: Naididae) to heavy contamination of sediments with polycyclic aromatic hydrocarbons. Sci. Total Environ. 827. doi: 10.1016/j.scitotenv.2022.153969, PMID: 35245562

[ref53] Jamal D. Penninckx M. J. (1999). Nitrification and Autotrophic Nitrifying Bacteria in a Hydrocarbon-Polluted Soil. Appl. Environ. Microbiol. 65, 4008–4013. doi: 10.1128/AEM.65.9.4008-4013.1999, PMID: 10473409 PMC99734

[ref54] Jesus H. E. Peixoto R. S. Rosado A. S. (2015). Bioremediation in Antarctic soils. J. Pet. Environ. Biotechnol. 6:2. doi: 10.4172/2157-7463.1000248

[ref55] Johnsen A. R. Boe U. S. Henriksen P. Malmquist L. M. V. Christensen J. H. (2021). Full-scale bioremediation of diesel-polluted soil in an Arctic landfarm. Environ. Pollut. 280:116946. doi: 10.1016/j.envpol.2021.116946, PMID: 33780839

[ref128] Josie V. D. Siciliano S. D. Winsley T. Snape I. Ferrari B. C. (2014). Bacterial targets as potential indicators of diesel fuel toxicity in subantarctic soils. Appl. Environ. Microbiol. 80, 4021–4033. doi: 10.1128/AEM.03939-13, PMID: 24771028 PMC4054227

[ref56] Jung S. W. Kang J. Park J. S. Joo H. M. Suh S.-S. Kang D.. (2021). Dynamic bacterial community response to *Akashiwo sanguinea* (Dinophyceae) bloom in indoor marine microcosms. Sci. Rep. 11:6983. doi: 10.1038/s41598-021-86590-8 33772091 PMC7997919

[ref57] Jung J. Yeom J. Kim J. Han J. Lim H. S. Park H.. (2011). Change in gene abundance in the nitrogen biogeochemical cycle with temperature and nitrogen addition in Antarctic soils. Res. Microbiol. 162, 1018–1026. doi: 10.1016/j.resmic.2011.07.007, PMID: 21839168

[ref58] Jurelevicius D. Alvarez V. M. Peixoto R. Rosado A. S. Seldin L. (2012). Bacterial polycyclic aromatic hydrocarbon ring-hydroxylating dioxygenases (PAH-RHD) encoding genes in different soils from King George Bay, Antarctic Peninsula. Appl. Soil Ecol. 55, 1–9. doi: 10.1016/j.apsoil.2011.12.008

[ref59] Jurelevicius D. Pereira R. D. S. da Mota F. F. Cury J. C. de Oliveira I. C. Rosado A. S.. (2021). Metagenomic analysis of microbial communities across a transect from low to highly hydrocarbon-contaminated soils in King George Island, Maritime Antarctica. Geobiology 20, 98–111. doi: 10.1111/gbi.12472 34545693

[ref60] Kai E. X. Wan Johari W. L. Habib S. Yasid N. A. Ahmad S. A. Shukor M. Y. (2020). The growth of the Rhodococcus sp. On diesel fuel under the effect of heavy metals and different concentrations of zinc. Advances. Pol. Sci. 31, 132–136. doi: 10.13679/j.advps.2019.0043

[ref61] Kauppi S. Sinkkonen A. Romantschuk M. (2011). Enhancing bioremediation of diesel-fuel-contaminated soil in a boreal climate: Comparison of biostimulation and bioaugmentation. Int. Biodeterior. Biodegradation 65, 359–368. doi: 10.1016/j.ibiod.2010.10.011

[ref62] Khomenkov V. G. Shevelev A. B. Zhukov V. G. Zagustina N. A. Bezborodov A. M. Popov V. O. (2008). Organization of metabolic pathways and molecular-genetic mechanisms of xenobiotic degradation in microorganisms: A review. Appl. Biochem. Microbiol. 44, 117–135. doi: 10.1134/S0003683808020014, PMID: 18669255

[ref63] Koshlaf E. Shahsavari E. Haleyur N. Mark Osborn A. Ball A. S. (2019). Effect of biostimulation on the distribution and composition of the microbial community of a polycyclic aromatic hydrocarbon-contaminated landfill soil during bioremediation. Geoderma 338, 216–225. doi: 10.1016/j.geoderma.2018.12.001

[ref64] Kuc V. Vázquez S. Hernández E. Martinez-Alvarez L. Villalba Primitz J. Mac Cormack W. P.. (2019). Hydrocarbon-contaminated Antarctic soil: changes in bacterial community structure during the progress of enrichment cultures with different n-alkanes as substrate. Polar Biol. 42, 1157–1166. doi: 10.1007/s00300-019-02508-1

[ref65] Kumar S. Suyal D. C. Yadav A. Shouche Y. Goel R. (2020). Psychrophilic Pseudomonas helmanticensis proteome under simulated cold stress. Cell Stress Chaperones 25, 1025–1032. doi: 10.1007/s12192-020-01139-4, PMID: 32683538 PMC7591641

[ref66] Lamas A. Regal P. Vázquez B. Miranda J. M. Franco C. M. Cepeda A. (2019). Transcriptomics: a powerful tool to evaluate the behavior of foodborne pathogens in the food production chain. Food Res. Int. 125:108543. doi: 10.1016/j.foodres.2019.108543, PMID: 31554082

[ref67] Lasa A. di Cesare A. Tassistro G. Borello A. Gualdi S. Furones D.. (2019). Dynamics of the Pacific oyster pathobiota during mortality episodes in Europe assessed by 16S rRNA gene profiling and a new target enrichment next-generation sequencing strategy. Environ. Microbiol. 21, 4548–4562. doi: 10.1111/1462-2920.14750, PMID: 31325353 PMC7379488

[ref68] Leite M. F. A. van den Broek S. W. E. B. Kuramae E. E. (2022). Current Challenges and Pitfalls in Soil Metagenomics. Microorganisms 10:1900. doi: 10.3390/microorganisms10101900, PMID: 36296177 PMC9606969

[ref69] Li S.-W. Huang Y.-X. Liu M.-Y. (2020). Transcriptome profiling reveals the molecular processes for survival of *Lysinibacillus fusiformis* strain 15-4 in petroleum environments. Ecotoxicol. Environ. Saf. 192:250. doi: 10.1016/j.ecoenv.2020.110250, PMID: 32028154

[ref70] Li J. Xu Y. Song Q. Zhang S. Xie L. Yang J. (2021). Transmembrane transport mechanism of n-hexadecane by *Candida tropicalis*: Kinetic study and proteomic analysis. Ecotoxicol. Environ. Saf. 209:111789. doi: 10.1016/j.ecoenv.2020.111789, PMID: 33340957

[ref71] Li B. Yan T. (2021). Next generation sequencing reveals limitation of qPCR methods in quantifying emerging antibiotic resistance genes (ARGs) in the environment. Appl. Microbiol. Biotechnol. 105, 2925–2936. doi: 10.1007/s00253-021-11202-4, PMID: 33738553

[ref72] Ling H. Hou J. Du M. Zhang Y. Liu W. Christie P.. (2023). Surfactant-enhanced bioremediation of petroleum-contaminated soil and microbial community response: A field study. Chemosphere 322:138225. doi: 10.1016/j.chemosphere.2023.138225, PMID: 36828103

[ref73] Liu Y.-F. Qi Z.-Z. Shou L.-B. Liu J.-F. Yang S.-Z. Gu J.-D.. (2019). Anaerobic hydrocarbon degradation in candidate phylum ‘Atribacteria’ (JS1) inferred from genomics. ISME J. 13, 2377–2390. doi: 10.1038/s41396-019-0448-2, PMID: 31171858 PMC6776118

[ref74] Lv Y. Bao J. Liu D. Gao X. Yu Y. Zhu L. (2023). Synergistic effects of rice husk biochar and aerobic composting for heavy oil-contaminated soil remediation and microbial community succession evaluation. J. Hazard. Mater. 448:130929. doi: 10.1016/j.jhazmat.2023.130929, PMID: 36860035

[ref75] Macchi M. Festa S. Nieto E. Irazoqui J. M. Vega-Vela N. E. Junca H.. (2021). Design and evaluation of synthetic bacterial consortia for optimized phenanthrene degradation through the integration of genomics and shotgun proteomics. Biotechnol Reports 29:e00588. doi: 10.1016/j.btre.2021.e00588, PMID: 33489789 PMC7809168

[ref76] Madison A. S. Sorsby S. J. Wang Y. Key T. A. (2023). Increasing in situ bioremediation effectiveness through field-scale application of molecular biological tools. Front. Microbiol. 13. doi: 10.3389/fmicb.2022.1005871, PMID: 36845972 PMC9950576

[ref77] Magalhães C. M. Machado A. Frank-Fahle B. Lee C. K. Cary S. C. (2014). The ecological dichotomy of ammonia-oxidizing archaea and bacteria in the hyper-arid soils of the Antarctic Dry Valleys. Front. Microbiol. 5:515. doi: 10.3389/fmicb.2014.00515, PMID: 25324835 PMC4179728

[ref78] Manuel K. (2022). Metaproteomics: Much More than Measuring Gene Expression in Microbial Communities. MSystems 4, e00115–e00119. doi: 10.1128/mSystems.00115-19 PMC6529545 31117019

[ref79] Martínez Álvarez L. M. Lo Balbo A. Mac Cormack W. P. Ruberto L. A. M. (2015). Bioremediation of a petroleum hydrocarbon-contaminated Antarctic soil: Optimization of a biostimulation strategy using response-surface methodology (RSM). Cold Reg. Sci. Technol. 119, 61–67. doi: 10.1016/j.coldregions.2015.07.005

[ref80] Martínez Álvarez L. M. Ruberto L. A. M. Lo Balbo A. Mac Cormack W. P. (2017). Bioremediation of hydrocarbon-contaminated soils in cold regions: Development of a pre-optimized biostimulation biopile-scale field assay in Antarctica. Sci. Total Environ. 590–591, 194–203. doi: 10.1016/j.scitotenv.2017.02.204 28262358

[ref81] Méndez García M. García de Llasera M. P. (2021). A review on the enzymes and metabolites identified by mass spectrometry from bacteria and microalgae involved in the degradation of high molecular weight PAHs. Sci. Total Environ. 797:149035. doi: 10.1016/j.scitotenv.2021.149035, PMID: 34303250

[ref82] Meziti A. Rodriguez-R L. M. Hatt J. K. Peña-Gonzalez A. Levy K. Konstantinidis K. T. (2021). The Reliability of Metagenome-Assembled Genomes (MAGs) in Representing Natural Populations: Insights from Comparing MAGs against Isolate Genomes Derived from the Same Fecal Sample. Appl. Environ. Microbiol. 87, e02593–e02520. doi: 10.1128/AEM.02593-20 33452027 PMC8105024

[ref83] Meziti A. Tsementzi D. Rodriguez-R L. M. Hatt J. K. Karayanni H. Kormas K. A.. (2019). Quantifying the changes in genetic diversity within sequence-discrete bacterial populations across a spatial and temporal riverine gradient. ISME J. 13, 767–779. doi: 10.1038/s41396-018-0307-6, PMID: 30397261 PMC6461791

[ref84] Mills D. K. Fitzgerald K. Litchfield C. D. Gillevet P. M. (2003). A comparison of DNA profiling techniques for monitoring nutrient impact on microbial community composition during bioremediation of petroleum-contaminated soils. J. Microbiol. Methods 54, 57–74. doi: 10.1016/S0167-7012(03)00007-1, PMID: 12732422

[ref85] Mohn W. Radziminski C. Fortin M.-C. Reimer K. (2001). On site bioremediation of hydrocarbon-contaminated Arctic tundra soils in inoculated biopiles. Appl. Microbiol. Biotechnol. 57, 242–247. doi: 10.1007/s002530100713 11693928

[ref86] Muangchinda C. Chavanich S. Viyakarn V. Watanabe K. Imura S. Vangnai A. S.. (2015). Abundance and diversity of functional genes involved in the degradation of aromatic hydrocarbons in Antarctic soils and sediments around Syowa Station. Environ. Sci. Pollut. Res. 22, 4725–4735. doi: 10.1007/s11356-014-3721-y, PMID: 25335763

[ref87] Mukherjee A. K. Chanda A. Mukherjee I. Kumar P. (2022). Characterization of lipopeptide biosurfactant produced by a carbazole-degrading bacterium *Roseomonas cervicalis*: The role of biosurfactant in carbazole solubilisation. J. Appl. Microbiol. 132, 1062–1078. doi: 10.1111/jam.15258, PMID: 34415661

[ref88] Nelson W. C. Tully B. J. Mobberley J. M. (2020). Biases in genome reconstruction from metagenomic data. PeerJ 8:e10119. doi: 10.7717/peerj.10119, PMID: 33194386 PMC7605220

[ref89] Païssé S. Goñi-Urriza M. Coulon F. Duran R. (2010). How a bacterial community originating from a contaminated coastal sediment responds to an oil input. Microb. Ecol. 60, 394–405. doi: 10.1007/s00248-010-9721-7, PMID: 20652237

[ref90] Parkavi K. Raja R. Arunkumar K. Coelho A. Hemaiswarya S. Carvalho I. S. (2020). “Recent Insights Into Algal Biotechnology” in Encyclopedia of Marine Biotechnology. ed. Kim S.-K. (Hoboken, NJ: Wiley)

[ref91] Parrilli E. Papa R. Tutino M. L. Sannia G. (2010). Engineering of a psychrophilic bacterium for the bioremediation of aromatic compounds. Bioeng. Bugs 1, 213–216. doi: 10.4161/bbug.1.3.11439 21326928 PMC3026427

[ref92] Paul C. (2022). A “Cultural” Renaissance: genomics breathes new life into an old craft. MSystems 4, e00092–e00019. doi: 10.1128/mSystems.00092-19 PMC6533372 31219785

[ref93] Pawar R. M. (2012). The effect of soil pH on degradation of polycyclic aromatic hydrocarbons (PAHs). Lahore: University of Hertfordshire.

[ref95] Powell S. M. Ferguson S. H. Snape I. Siciliano S. D. (2006). Fertilization stimulates anaerobic fuel degradation of antarctic soils by denitrifying microorganisms. Environ. Sci. Technol. 40, 2011–2017. doi: 10.1021/es051818t, PMID: 16570629

[ref96] Pudasaini S. Wilkins D. Adler L. Hince G. Spedding T. King C.. (2019). Characterization of polar metabolites and evaluation of their potential toxicity in hydrocarbon contaminated Antarctic soil elutriates. Sci. Total Environ. 689, 390–397. doi: 10.1016/j.scitotenv.2019.06.389, PMID: 31277006

[ref97] Ramage J. Jungsberg L. Wang S. Westermann S. Lantuit H. Heleniak T. (2021). Population living on permafrost in the Arctic. Popul. Environ. 43, 22–38. doi: 10.1007/s11111-020-00370-6, PMID: 35882324

[ref98] Ramírez-Fernández L. Orellana L. H. Johnston E. R. Konstantinidis K. T. Orlando J. (2021). Diversity of microbial communities and genes involved in nitrous oxide emissions in Antarctic soils impacted by marine animals as revealed by metagenomics and 100 metagenome-assembled genomes. Sci. Total Environ. 788:147693. doi: 10.1016/j.scitotenv.2021.147693, PMID: 34029816

[ref99] Rao R. (2003). From unstructured data to actionable intelligence. IT Professional 5, 29–35. doi: 10.1109/MITP.2003.1254966, PMID: 36974740

[ref100] Revill A. T. Snape I. Lucieer A. Guille D. (2007). Constraints on transport and weathering of petroleum contamination at Casey Station, Antarctica. Cold Regions Sci. Technol. 48, 154–167. doi: 10.1016/j.coldregions.2007.01.001

[ref101] Rio D. C. (2014). Reverse transcription–polymerase chain reaction. Cold Spring Harb. Protoc. 2014:pdb.prot080887. doi: 10.1101/pdb.prot080887, PMID: 25368309

[ref102] Ruberto L. Dias R. Lo Balbo A. Vazquez S. C. Hernandez E. A. Mac Cormack W. P. (2009). Influence of nutrients addition and bioaugmentation on the hydrocarbon biodegradation of a chronically contaminated Antarctic soil. J. Appl. Microbiol. 106, 1101–1110. doi: 10.1111/j.1365-2672.2008.04073.x, PMID: 19191978

[ref103] Ruberto L. Vazquez S. C. Dias R. L. Hernández E. A. Coria S. H. Levin G.. (2010). Small-scale studies towards a rational use of bioaugmentation in an Antarctic hydrocarbon-contaminated soil. Antarct. Sci. 22, 463–469. doi: 10.1017/S0954102010000295

[ref104] Ruberto L. A. M. Vazquez S. Lobalbo A. Mac Cormack W. P. (2005). Psychrotolerant hydrocarbon-degrading Rhodococcus strains isolated from polluted Antarctic soils. Antarct. Sci. 17, 47–56. doi: 10.1017/S0954102005002415

[ref105] Ruberto L. Vazquez S. C. Mac Cormack W. P. (2003). Effectiveness of the natural bacterial flora, biostimulation and bioaugmentation on the bioremediation of a hydrocarbon contaminated Antarctic soil. Int. Biodeterior. Biodegradation 52, 115–125. doi: 10.1016/S0964-8305(03)00048-9, PMID: 19191978

[ref106] Ruberto L. A. M. Vázquez S. C. Mac Cormack W. P. (2009). Bacterial hydrocarbon-degrading consortium from Antarctic soils. Revista Argentina de Microbiologia 41:262. PMID: 20085191

[ref107] Rushimisha I. E. Li X. Han T. Chen X. Wang K. Weng L.. (2023). Effect of fresh and aged biochar on electrogenic hydrocarbon degradation in soil microbial electrochemical remediation. Electrochim. Acta 440:141713. doi: 10.1016/j.electacta.2022.141713

[ref108] Sampaio D. S. Almeida J. R. B. de Jesus H. E. Rosado A. S. Seldin L. Jurelevicius D. (2017). Distribution of anaerobic hydrocarbon-degrading bacteria in soils from king George Island, Maritime Antarctica. Microbial Ecol. 74, 810–820. doi: 10.1007/s00248-017-0973-3, PMID: 28484799

[ref109] Sansupa C. Wahdan S. F. M. Hossen S. Disayathanoowat T. Wubet T. Purahong W. (2021). Can we use functional annotation of prokaryotic taxa (FAPROTAX) to assign the ecological functions of soil bacteria? Appl. Sci. 11:688. doi: 10.3390/app11020688

[ref110] Schenk S. Bannister S. C. Sedlazeck F. J. Anrather D. Minh B. Q. Bileck A.. (2019). Combined transcriptome and proteome profiling reveals specific molecular brain signatures for sex, maturation and circalunar clock phase. elife 8:e41556. doi: 10.7554/eLife.41556, PMID: 30767890 PMC6377233

[ref111] Semenova E. M. Babich T. L. Sokolova D. S. Ershov A. P. Raievska Y. I. Bidzhieva S. K.. (2022). Microbial communities of seawater and coastal soil of Russian Arctic Region and their potential for bioremediation from Hydrocarbon Pollutants. Microorganisms 10:1490. doi: 10.3390/microorganisms10081490, PMID: 35893548 PMC9332119

[ref112] Shah S. H. H. Lei S. Ali M. Doronin D. Hussain S. T. (2020). Prosumption: bibliometric analysis using HistCite and VOSviewer. Kybernetes 49, 1020–1045. doi: 10.1108/K-12-2018-0696

[ref113] Shahroodi T. Zahedi M. Singh A. Wong S. Hamdioui S. (2022). KrakenOnMem: A Memristor-Augmented HW/SW Framework for Taxonomic Profiling. *Proceedings of the 36th ACM International Conference on Supercomputing*.

[ref114] Silva R. Padovani K. Góes F. Alves R. (2021). geneRFinder: gene finding in distinct metagenomic data complexities. BMC Bioinformatics 22:87. doi: 10.1186/s12859-021-03997-w 33632132 PMC7905635

[ref115] Silva-Castro G. A. Rodelas B. Perucha C. Laguna J. González-López J. Calvo C. (2013). Bioremediation of diesel-polluted soil using biostimulation as post-treatment after oxidation with Fenton-like reagents: Assays in a pilot plant. Sci. Total Environ. 445–446, 347–355. doi: 10.1016/j.scitotenv.2012.12.081 23354375

[ref116] Simas F. N. B. Schaefer C. E. G. R. Michel R. F. M. Francelino M. R. Bockheim J. G. (2015). “Soils of the South Orkney and South Shetland Islands, Antarctica” in The Soils of Antarctica. ed. Bockheim J. G. (New York: Springer International Publishing), 227–273.

[ref117] Stallwood B. Shears J. Williams P. A. Hughes K. A. (2005). Low temperature bioremediation of oil-contaminated soil using biostimulation and bioaugmentation with a Pseudomonas sp. from maritime Antarctica. J. Appl. Microbiol. 99, 794–802. doi: 10.1111/j.1365-2672.2005.02678.x, PMID: 16162230

[ref118] Su Z. Wang S. Yang S. Yin Y. Cao Y. Li G.. (2022). Genetic and comparative genome analysis of *Exiguobacterium aurantiacum* SW-20, a petroleum-degrading bacteria with salt tolerance and heavy metal-tolerance isolated from produced water of Changqing oilfield, China. Microorganisms 10:66. doi: 10.3390/microorganisms10010066, PMID: 35056515 PMC8779447

[ref119] Sui X. Wang X. Yu L. Ji H. (2023). Genomics for the characterization of the mechanisms of microbial strains in degrading petroleum pollutants. Environ. Sci. Pollut. Res. 30, 21608–21618. doi: 10.1007/s11356-022-23685-3, PMID: 36271069

[ref120] Tikariha H. Purohit H. J. (2019). Assembling a genome for novel nitrogen-fixing bacteria with capabilities for utilization of aromatic hydrocarbons. Genomics 111, 1824–1830. doi: 10.1016/j.ygeno.2018.12.005, PMID: 30552976

[ref121] Tin T. Fleming Z. L. Hughes K. A. Ainley D. G. Convey P. Moreno C. A.. (2009). Impacts of local human activities on the Antarctic environment. Antarct. Sci. 21, 3–33. doi: 10.1017/S0954102009001722, PMID: 36611770

[ref122] Tribelli P. M. Rossi L. Ricardi M. M. Gomez-Lozano M. Molin S. Raiger Iustman L. J.. (2018). Microaerophilic alkane degradation in *Pseudomonas extremaustralis*: a transcriptomic and physiological approach. J. Ind. Microbiol. Biotechnol. 45, 15–23. doi: 10.1007/s10295-017-1987-z, PMID: 29116430

[ref123] Tschitschko B. Williams T. J. Allen M. A. Zhong L. Raftery M. J. Cavicchioli R. (2016). Ecophysiological Distinctions of Haloarchaea from a Hypersaline Antarctic Lake as Determined by Metaproteomics. Appl. Environ. Microbiol. 82, 3165–3173. doi: 10.1128/AEM.00473-16, PMID: 26994078 PMC4959232

[ref124] Tucci M. Viggi C. C. Crognale S. Matturro B. Rossetti S. Capriotti A. L.. (2022). Insights into the syntrophic microbial electrochemical oxidation of toluene: a combined chemical, electrochemical, taxonomical, functional gene-based, and metaproteomic approach. Sci. Total Environ. 850:157919. doi: 10.1016/j.scitotenv.2022.157919, PMID: 35964739

[ref125] Tveit A. T. Urich T. Svenning M. M. (2014). Metatranscriptomic Analysis of Arctic Peat Soil Microbiota. Appl. Environ. Microbiol. 80, 5761–5772. doi: 10.1128/AEM.01030-14, PMID: 25015892 PMC4178616

[ref126] Ugarte A. Quang-Dao M. Ho B. H. Vigliotti C. Belda E. Zucker J.-D.. (2019). QMSpy: an Integrated Modular and Scalable Platform for Quantitative Metagenomics in Pyspark. *2019 IEEE-RIVF International Conference on Computing and Communication Technologies (RIVF), 1–6*.

[ref127] van Dorst J. Bissett A. Palmer A. S. Brown M. Snape I. Stark J. S.. (2014). Community fingerprinting in a sequencing world. FEMS Microbiol. Ecol. 89, 316–330. doi: 10.1111/1574-6941.12308, PMID: 24580036

[ref129] van Dorst J. Wilkins D. Crane S. Montgomery K. Zhang E. Spedding T.. (2021). Microbial community analysis of biopiles in Antarctica provides evidence of successful hydrocarbon biodegradation and initial soil ecosystem recovery. Environ. Pollut. 290:117977. doi: 10.1016/j.envpol.2021.117977, PMID: 34416497

[ref130] van Dorst J. Wilkins D. King C. K. Spedding T. Hince G. Zhang E.. (2020). Applying microbial indicators of hydrocarbon toxicity to contaminated sites undergoing bioremediation on subantarctic Macquarie Island. Environ. Pollut. 259:113780. doi: 10.1016/j.envpol.2019.113780, PMID: 31887587

[ref131] van Eck N. J. Waltman L. (2011). Text mining and visualization using VOSviewer. ArXiv abs/1109.2058. doi: 10.48550/arXiv.1109.2058

[ref132] Van Goethem M. W. Vikram S. Hopkins D. W. Hall G. Woodborne S. Aspray T. J.. (2020). Nutrient parsimony shapes diversity and functionality in hyper-oligotrophic Antarctic soils. BioRxiv 2020:15.950717. doi: 10.1101/2020.02.15.950717

[ref133] von Wintzingerode F. Böcker S. Schlötelburg C. Chiu N. H. L. Storm N. Jurinke C.. (2002). Base-specific fragmentation of amplified 16S rRNA genes analyzed by mass spectrometry: A tool for rapid bacterial identification. Proc. Natl. Acad. Sci. 99, 7039–7044. doi: 10.1073/pnas.102165899, PMID: 11983869 PMC124524

[ref134] Wang Y. Hou Y. Wang Q. Wang Y. (2021). The elucidation of the biodegradation of nitrobenzene and p-nitrophenol of nitroreductase from Antarctic psychrophile Psychrobacter sp. ANT206 under low temperature. J. Hazard. Mater. 413. doi: 10.1016/j.jhazmat.2021.125377, PMID: 33609870

[ref135] Wang Q. Zhu R. Zheng Y. Bao T. Hou L. (2019). Effects of sea animal colonization on the coupling between dynamics and activity of soil ammonia-oxidizing bacteria and archaea in maritime Antarctica. Biogeosciences 16, 4113–4128. doi: 10.5194/bg-16-4113-2019

[ref136] Waschulin V. Borsetto C. James R. Newsham K. K. Donadio S. Corre C.. (2022). Biosynthetic potential of uncultured Antarctic soil bacteria revealed through long-read metagenomic sequencing. ISME J. 16, 101–111. doi: 10.1038/s41396-021-01052-3, PMID: 34253854 PMC8692599

[ref137] Watahiki S. Kimura N. Yamazoe A. Miura T. Sekiguchi Y. Noda N.. (2019). Ecological impact assessment of a bioaugmentation site on remediation of chlorinated ethylenes by multi-omics analysis. J. Gen. Appl. Microbiol. 65, 225–233. doi: 10.2323/jgam.2018.10.003, PMID: 30853704

[ref138] Whelan M. J. Coulon F. Hince G. Rayner J. McWatters R. Spedding T.. (2015). Fate and transport of petroleum hydrocarbons in engineered biopiles in polar regions. Chemosphere 131, 232–240. doi: 10.1016/j.chemosphere.2014.10.088, PMID: 25563162

[ref139] Whyte L. G. Bourbonnière L. Bellerose C. Greer C. W. (1999). Bioremediation assessment of hydrocarbon-contaminated soils from the high Arctic. Biorem. J. 3, 69–80. doi: 10.1080/10889869991219217, PMID: 20199573

[ref140] Wilmes P. Bond P. L. (2006). Metaproteomics: studying functional gene expression in microbial ecosystems. Trends Microbiol. 14, 92–97. doi: 10.1016/j.tim.2005.12.006, PMID: 16406790

[ref141] Wong R. R. Lim Z. S. Shaharuddin N. A. Zulkharnain A. Gomez-Fuentes C. Ahmad S. A. (2021). Diesel in Antarctica and a bibliometric study on its indigenous microorganisms as remediation agent. Int. J. Environ. Res. Public Health 18:1512. doi: 10.3390/ijerph18041512, PMID: 33562609 PMC7915771

[ref142] Xue S. Huang C. Tian Y. Li Y. Li J. Ma Y. (2020). Synergistic effect of Rhamnolipids and inoculation on the bioremediation of petroleum-contaminated soils by bacterial consortia. Curr. Microbiol. 77, 997–1005. doi: 10.1007/s00284-020-01899-3, PMID: 32002627

[ref143] Yergeau E. Sanschagrin S. Beaumier D. Greer C. W. (2012). Metagenomic Analysis of the Bioremediation of Diesel-Contaminated Canadian High Arctic Soils. PLoS One 7:e30058. doi: 10.1371/journal.pone.0030058 22253877 PMC3256217

[ref144] Zhang E. Czechowski P. Terauds A. Wong S. Y. Chelliah D. S. Raven M.. (2021). Tracing boundaries in Eastern Antarctica: Multi-scale drivers of soil microbial communities across the hyperarid Vestfold Hills. BioRxiv 2021:22.461446. doi: 10.1101/2021.09.22.461446

[ref145] Zhang S. Hu Z. Wang H. (2019). Metagenomic analysis exhibited the co-metabolism of polycyclic aromatic hydrocarbons by bacterial community from estuarine sediment. Environ. Int. 129, 308–319. doi: 10.1016/j.envint.2019.05.028, PMID: 31150973

[ref146] Zhang L. Li X. Zuo W. Li S. Sun G. Wang W.. (2021). Root exuded low-molecular-weight organic acids affected the phenanthrene degrader differently: a multi-omics study. J. Hazard. Mater. 414. doi: 10.1016/j.jhazmat.2021.125367, PMID: 33677320

[ref147] Zhang Z. Lo I. M. C. (2015). Biostimulation of petroleum-hydrocarbon-contaminated marine sediment with co-substrate: involved metabolic process and microbial community. Appl. Microbiol. Biotechnol. 99, 5683–5696. doi: 10.1007/s00253-015-6420-9, PMID: 25661814

[ref148] Zhang L. Wang M. Cui H. Qiao J. Guo D. Wang B.. (2022). How humic acid and Tween80 improve the phenanthrene biodegradation efficiency: insight from cellular characteristics and quantitative proteomics. J. Hazard. Mater. 421:126685. doi: 10.1016/j.jhazmat.2021.126685, PMID: 34332485

[ref149] Zhao B. Poh C. L. (2008). Insights into environmental bioremediation by microorganisms through functional genomics and proteomics. Proteomics 8, 874–881. doi: 10.1002/pmic.200701005, PMID: 18210372

[ref150] Zhu R. Liu Y. Xu H. Ma D. Jiang S. (2013). Marine animals significantly increase tundra N2O and CH4 emissions in maritime Antarctica. J. Geophys. Res. Biogeo. 118, 1773–1792. doi: 10.1002/2013JG002398

